# A 3′-5′ exonuclease activity embedded in the helicase core domain of *Candida albicans* Pif1 helicase

**DOI:** 10.1038/srep42865

**Published:** 2017-02-20

**Authors:** Xiao-Bin Wei, Bo Zhang, Nicolas Bazeille, Ying Yu, Na-Nv Liu, Brigitte René, Olivier Mauffret, Xu-Guang Xi

**Affiliations:** 1 College of Life Sciences, Northwest A&F University, Yangling, Shaanxi 712100, China; 2 LBPA, ENS-Cachan, CNRS, Université Paris-Saclay, 61 Avenue du Président Wilson, 94235 Cachan, France

## Abstract

3′-5′ exonucleases are frequently found to be associated to polymerases or helicases domains in the same enzyme or could function as autonomous entities. Here we uncovered that *Candida albicans* Pif1 (CaPif1) displays a 3′-5′ exonuclease activity besides its main helicase activity. These two latter activities appear to reside on the same polypeptide and the new exonuclease activity could be mapped to the helicase core domain. We clearly show that CaPif1 displays exclusively exonuclease activity and unambiguously establish the directionality of the exonuclease activity as the 3′-to-5′ polarity. The enzyme appears to follow the two-metal-ion driven hydrolyzing activity exhibited by most of the nucleases, as shown by its dependence of magnesium and also by the identification of aspartic residues. Interestingly, an excellent correlation could be found between the presence of the conserved residues and the exonuclease activity when testing activities on Pif1 enzymes from eight fungal organisms. In contrast to others proteins endowed with the double helicase/exonuclease functionality, CaPif1 differs in the fact that the two activities are embedded in the same helicase domain and not located on separated domains. Our findings may suggest a biochemical basis for mechanistic studies of Pif1 family helicases.

Nucleases cleave the phosphodiester bonds of nucleic acids and play essential roles in many aspects of cellular metabolism and in the defense against viral invasions^1^,^2^. While endonucleases cut nucleic acids internally, yielding poly or oligonucleotides, exonucleases catalyze the excision of nucleotide monophosphates (dNMPs) from 3′-or 5′-DNA termini. Exonucleases can be classified into several classes according to their functional and conformational states: autonomous exonucleases, polymerases-associated exonucleases, and helicases-associated exonuclases^1^.

One of the main examples of helicase-associated exonuclease is the RecBCD enzyme: a heterotrimeric helicase/nuclease that initiates homologous recombination at double-stranded DNA breaks^3^. The enzyme is driven by two motor subunits, RecB and RecD, translocating on opposite single-strands of the DNA duplex^4^,^5^. During unwinding, the nuclease in RecB domain nicks 3′-strand or cleaves both DNA strands endonucleolytically depending on the Mg^2+^ concentration^6^. Another example is the protein involved in the Werner syndrome (WS), a genomic instability disorder characterized by premature aging symptoms^7^. The WRN gene defective in the WS encodes a protein of the RecQ helicase family possessing both 3′-5′ helicase at C-terminus and 3′-5′ exonuclease activities at the amino terminus in the same polypeptide^8^,^9^. While the WRN helicase and exonuclease domains are shown to act independently on D loops and Holliday junctions, the two domains could act in a coordinated fashion *in vitro* on a forked duplex^10^, or to resolve a recombination intermediate^10^,^11^.

Pif1 family of helicases is highly conserved from bacteria to human^12^. The interest for Pif1 helicases has been considerably increased since the discovery that this family of helicases plays multiple functional roles in the maintenance of genomic integrity: it disrupts telomerase from the telomere ends and advances fork progression at many nuclear sites; facilitates replication and suppresses DNA damage at G-quadruplex motifs; processes Okazaki fragments maturation in cooperation with Dna2 helicase/nuclease and promotes break-induced replication (BIR) via bubble migration^13^,^14^,^15^,^16^,^17^,^18^. In this study, we report a previously undiscovered helicase-associated exonuclease activity from CaPif1. Importantly, different from the above mentioned helicase-associated exonucleases in which the helicase and exonuclease domains possess structurally autonomous domains or motifs, and each helicase and exonuclease domain being active in isolation, the residues implicated in exonuclease active sites of CaPif1 appear to be embedded in Pif1 helicase domains, indicating that the helicase and exonuclease activities are not structurally separable. More interestingly, it appears that this property is well conserved, at least, among the fungal species.

## Results

### DNA helicase and DNA nuclease activities reside on the same polypeptide

**Figure 1:**
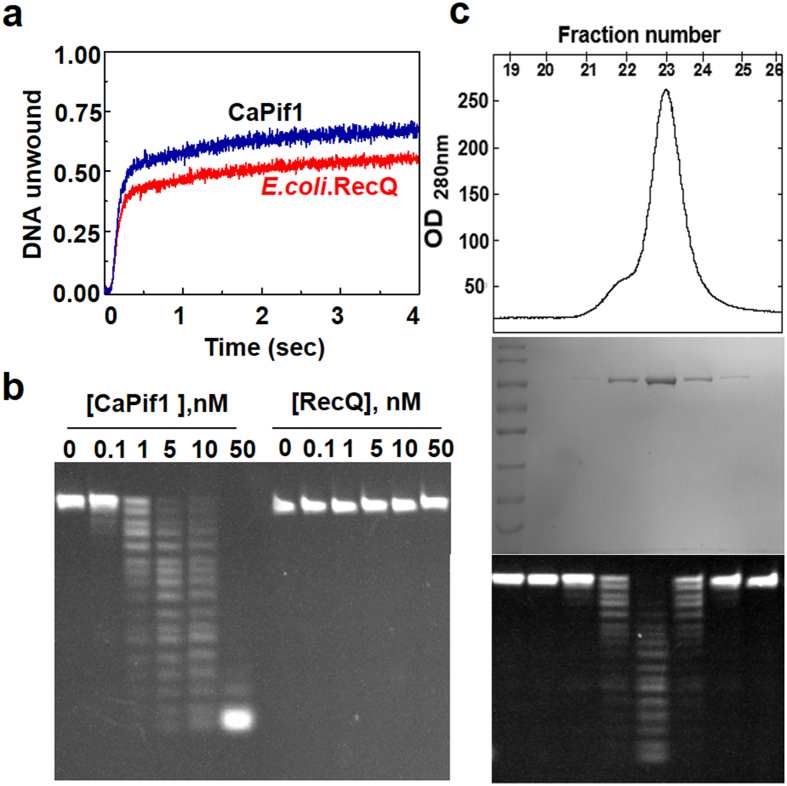
Helicase and exonuclease activities of purified CaPif1. (**a**) Purified *E. coli* RecQ and CaPif1-catalyzed partial duplex unwinding. The unwinding reaction was performed as described under Materials and Methods. 5 nM protein and 4 nM fluorescein/hexachlorofluorescein labeled DNA (Substrate K, Supplementary Table S1) were used in the assay. (**b**) Exonuclease activity of the purified proteins. Gel electrophoresis measurements of exonuclease activity of the both purified proteins. The reactions were carried out at 37 °C for 5 min in buffer A with 400 nM 5′-fluorescein labeled 20-nt ssDNA (substrate S^20^) at the indicated protein concentrations. (**c**) Copurification of CaPif1 and exonuclease activity. Purified CaPif1 by size-exclusion chromatography (up panel) was analyzed by SDS-PAGE and Coomassie blue staining (middle panel). Fractions 19–26 were used for nuclease assay under the standard assay condition (bottom panel).

During analysis of CaPif1 helicase-catalyzed DNA unwinding using electrophoretic mobility shift assay (EMSA), we found that in addition to the expected unwound ssDNA product, a significant degradation of fluorescein labeled oligonucleotides appeared in the bottom of gels, suggesting that CaPif1 protein could possess some nuclease activity. Since CaPif1 was expressed and purified from *E. coli* and knowing that *E. coli* contains several 3′ to 5′ exonucleases including exonuclease I, exonuclease VII, and RecJ, capable of degrading single-stranded DNA^19^, we performed additional experiments to address whether the observed nuclease activity is intrinsic to CaPif1, or just stems from a contaminating nuclease resulting from unspecific copurification. First, to assess that the observed exonuclease activity of CaPif1 protein does not come from purification contamination, both CaPif1 protein and *E. coli* RecQ helicase were purified with identical procedures including nickel affinity column, ion exchange and size-exclusion chromotography. The latter protein was used as a mock control in sake of RecQ being a helicase with very strong ssDNA binding affinity. We observed that while the purified CaPif1 helicase unwinds efficiently a partial duplex DNA and degrades 5′-fluorescein labeled 20-nt ssDNA, the purified *E. coli* RecQ helicase displays only unwinding activity, but no any detectable nuclease activity (Fig. 1a,b). Second, the nuclease activity was assayed with the individual fractions from the size-exclusion column. As seen in Fig. 1c, CaPif1 protein co-elutes with the exonuclease activity tested with 5′-fluorescein labeled ssDNA. The fact that the observed nuclease activity was only co-purified with CaPif1 protein, suggest that the questioned nuclease activity should not be due to contaminating nucleases. The stronger evidence which rule out the purification contamination was further shown by the mutations of the conserved residues in active site (see below section). Furthermore, DNA degradation was dependent on both the time of incubation and the concentration of added CaPif1 protein (Fig. 1b). These results do not unambiguously prove, but strongly indicate that CaPif1 possesses both nuclease and helicase activities located on the same polypeptide. This conclusion was further strengthened by the following series of experiments shown below.

### The CaPif1 possesses an exonuclease, but not an endonuclease activity

**Figure 2:**
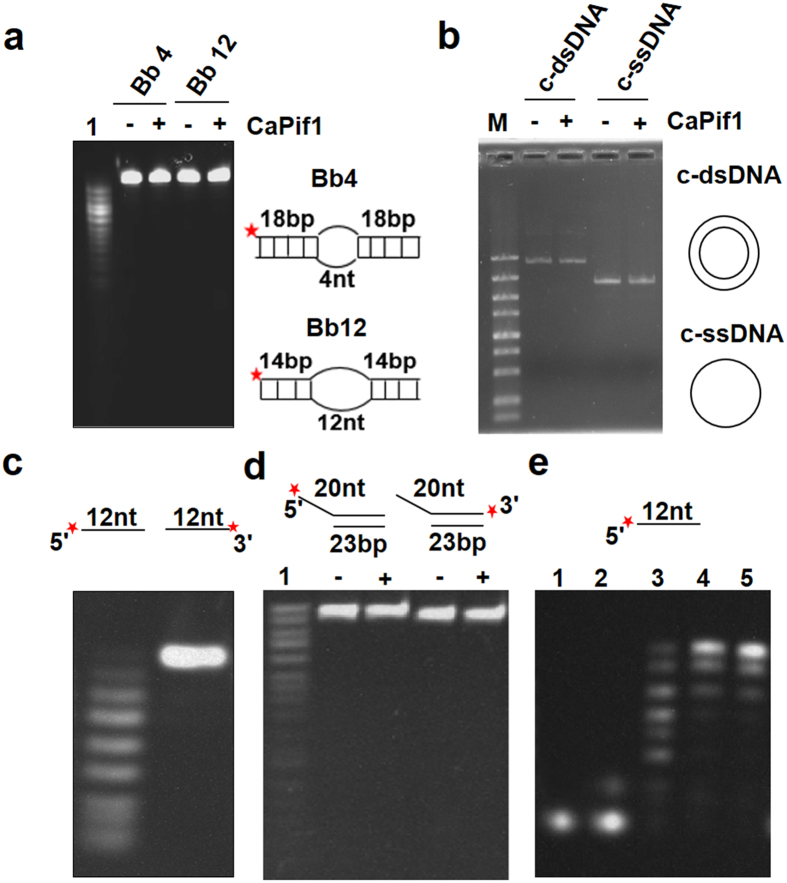
CaPif1 is a 3′-5′ exonuclease. (**a**) The prepared 400 nM Bubble DNAs (Bb^4^ and Bb^12^) were incubated with 1 nM CaPif1 for 5 min at 37 °C. The lane 1 is a control of 40-nt ssDNA. (**b**) Circular double stranded and single stranded DNAs were incubated with 500 nM CaPif1 for 5 min at 37 °C and analyzed with 1% agarose in buffer TBE. (**c**,**d**) The 5′-or 3′-labeled 12-nt oligos (substrate S^12^-5F or S^12^-3F) and 3′-or 5′- labeled partial duplex DNA (substrate S^20^D^23^-5F or S^20^D^23^-3F) were incubated with 1 nM CaPif1 for 5 min at 37 °C. (**e**) CaPif1 removes dNMP from the 3′-end of the substrate S^12^-5F with trinucleotides as the end product. The activity was assayed under standard condition with 20 nM CaPif1 and 400 nM DNA. Lane 1: fluorescein labeled ATP-γ-S; lane 2: completely digested substrate S^12^-5F; lanes 3–5: assays with increasing concentration of AMP from 1–3 mM.

Based on the fact that the cleavage products patterns were oligomers of gradually decreased sizes (Fig. 1b), we hypothesized that CaPif1 should be an exonuclease. To confirm this point, two bubble substrates with 4- and 12-nt unpaired bases inserted in the middle of blunt end dsDNA were used for DNA degradation assay. As expected, while the 5′-labeled partial single-stranded DNA (Lane 1 in Fig. 2a) was degraded to a series of smaller labeled products, both bubble substrates were not degraded at any extent under the same experimental conditions (Fig. 2a). Furthermore, consistently with the above observations, the circular forms of single (c-ssDNA) and double stranded DNAs (c-dsDNA) incubated with higher CaPif1 protein concentration (500 nM) for 30 min were not degraded (Fig. 2b). These data strongly suggested that CaPif1 displays an exonuclease, but not endonuclease activity.

### The CaPif1-Associated exonuclease exhibits a 3′-to-5′ polarity and possibly produces deoxynucleoside monophosphates

The directionality of the CaPif1-associated exonuclease was determined by using differently labeled ssDNA substrates. With the 5′-labeled substrate, the CaPif1-associated exonuclease produced a series of products with decreasing size (Fig. 2c). By contrast, digestion of 3′-labeled ssDNA did not give any hydrolyzed products due to the fact that the OH-group of ssDNA was blocked by dye group (Fig. 2c). These results strongly suggested that CaPif1-asssociated exonuclease possesses single strand exonuclease activity with a 3′-to-5′ polarity. To further confirm the 3′-to-5′ polarity of CaPif1, we used 5′-overhanged forked dsDNA in which fluorescein was labeled at 5′ or 3′ end, respectively (Fig. 2d). As expected, no exonuclease activity can be observed with the both substrates, confirming that CaPif1 only digests ssDNA in a 3′-to-5′ direction, but not a 5′-to-3′ direction. In accordance with this conclusion, when 5′-fluorescently labeled ssDNA was incubated with high concentration of CaPif1 300 nM for 1 hour, only a single major product migrates at the gel front, which corresponding to the complete digested fluoresceintly labeled 5′-end nucleotide (Fig. 2e, lane 2). This interpretation is confirmed by the co-migration of a fluorescein labeled ATP-γ-S with the completely digested labeled ssDNA (Fig. 2e, compare lane 1–2). Furthermore, this finding is in accordance with the results shown in Fig. 2e (lanes 3–5), in which the exonuclease activity is inhibited by increasing concentrations of nucleoside monophosphates, indicating that the excess amount of hydrolyzed products will bind and inhibit the active sites.

### Biochemical properties of the CaPif1-associated exonuclease activity

**Figure 3:**
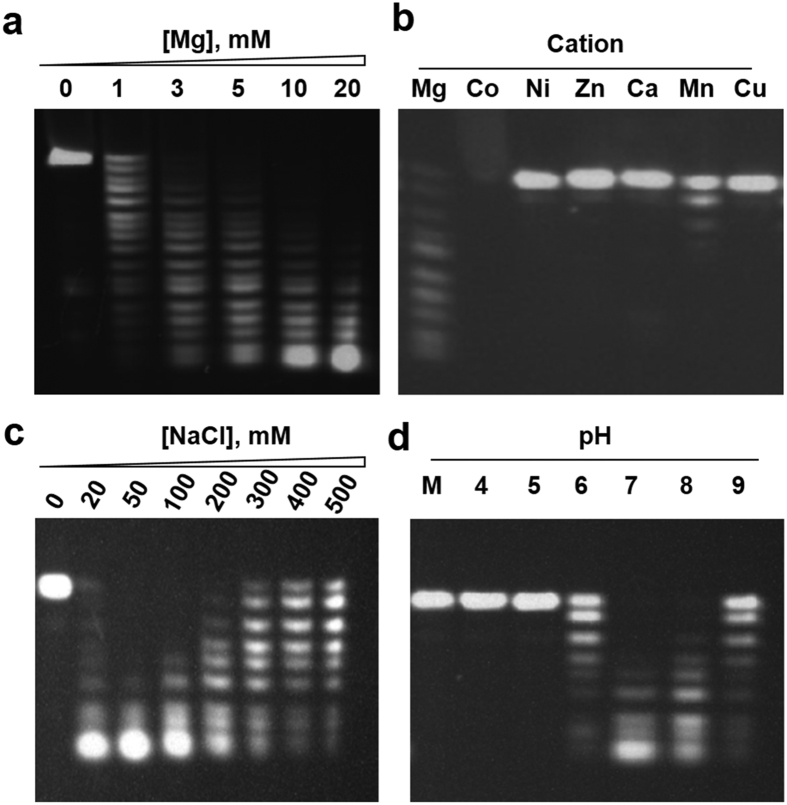
Characterization of CaPif1-associated exonuclease activity. All experiments were performed with 400 nM ssDNA (S^12^-5F) and 1 nM CaPif1 protein in buffer A and with the indicated Mg^2+^ concentration (**a**), 2 mM of different ions (**b**), NaCl concentration (**c**) and at different pH (**d**) in each panel. Reactions were performed at 37 °C for 5 min.

Usually, nucleases hydrolyze the phosphodiester linkages present in nucleic acids through a general one or two-metal-ion driven mechanism^20^,^21^. We first examined the effect of the ion nature on the CaPif1-catalyzed hydrolyzing activity. The CaPif1 exonuclease activity was found to be highly dependent of the Mg^2+^ concentration, where 1 mM was enough to ensure the significant activity, and the exonuclase activity increases as Mg^2+^ concentration increases (Fig. 3a). Mn^2+^ could only partially replace Mg^2+^ (Fig. 3b). With the same metal ions concentration (2 mM), Mn^2+^ gave rise to only 10–12% of the maximal activity seen with Mg^2+^ (compare the lane 1 and lane 6 in Fig. 3b). On the other hand, Co^2+^ induces precipitation of protein-DNA complex and consequently, only very faint signal can be detected. Furthermore, Zn^2+^, Ca^2+^, and Ni^2+^ could not replace Mg^2+^ or Mn^2+^ (Fig. 3b). DNA degradation was also shown to be sensitive to the salt addition, where NaCl exhibited optimum activity at ~50 mM and significant inhibition at ~500 mM (Fig. 3c). The pH optimum of the degradation reaction was at around pH 7–8, with 80% and 70% residual activity at pH values of 6.0 and 9.0, respectively (Fig. 3d). Furthermore, the exonuclease activity was inhibited almost completely by 5 mM GMP, a concentration that has been shown to inhibit effectively the 3′-to-5′ exonucleases activities of the mammalian DNA polymerases δ and ϵ and *E. coli* DNA polymerase I^22^. The CaPif1 associated exonuclease activity was similarly inhibited by dGMP, AMP, dAMP and CMP, but not by nucleoside triphosphates (Supplementary Fig. S1).

### CaPif1-associated exonuclease activity on different structured DNA substrate

**Figure 4:**
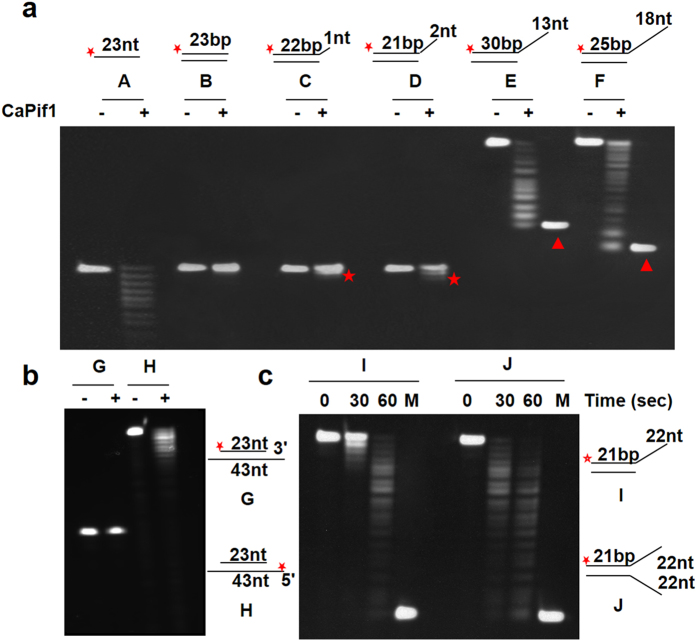
The substrates specificity of CaPif1-associated exonuclease activity. The DNA substrates used in the assays were schematically presented along with the gel. ((**a**), (**b**) and (**c**)). 400 nM labeled DNA and 1 nM CaPif1 protein were incubated in buffer A for 5 min at 37 °C. The DNAs were separated as described under the Materials and Methods. The red stars in figure (**a**) indicate the digested substrates C and D. The red triangles in figure (**a**) are size marker controls (30-nt and 25-nt, respectively).

To determine the type of DNA substrates preferentially degraded by CaPif1-associated exonuclease, we measured hydrolyzing activities of the protein with several designed DNA substrates. These latter were single stranded DNA, double-stranded blunt end DNA and partial duplex DNA with increasing length of ssDNA at 3′-end. These substrates were labeled at appropriate position with fluorescein as shown in Fig. 4 and incubated with 1 nM CaPif1 for 5 min. Measurements of the exonuclease activity of CaPif1 demonstrated that CaPif1 efficiently degrades 3′-protruding partial duplex DNA and leaves the blunt-end substrates undigested (Fig. 4a). This phenomenon was clearer with the 1 and 2 bases protruding at the DNA 3′-end (Fig. 4a, substrates C–D). However, CaPif1 was unable of digesting the blunt-end dsDNA (Fig. 4a, substrate B) and a recessed strand in a partial duplex DNA (Fig. 4b, substrate G). These results show that CaPif1-associated exonuclase could be distinguished from Werner exonuclease activity by its preference for ssDNA degradation while WRN exonuclease preferentially hydrolyzes the recessed strand in partial DNA duplexes^15^. To probe whether the 3′-5′ exonuclease activity could be affected by the presence of a non complementary 5′-ssDNA tail, we studied CaPif1-catalyzed DNA degradation of a 21-bp forked dsDNA with 22-nucleotide at 3′ and 5′-overhang, respectively (Fig. 4c). We found that CaPif1 degrades more efficiently the forked DNA than partial duplex DNA under the same experimental conditions (compare the results between the substrates I and J in Fig. 4c). These results altogether demonstrate that the ssDNA and a 3′-protruding single strand are preferential DNA substrates and that the presence of a non complementary strand could enhance CaPif1 associated exonuclease activity.

### The CaPif1-associated exonuclease activity maps to the helicase core domain of Pif1

**Figure 5:**
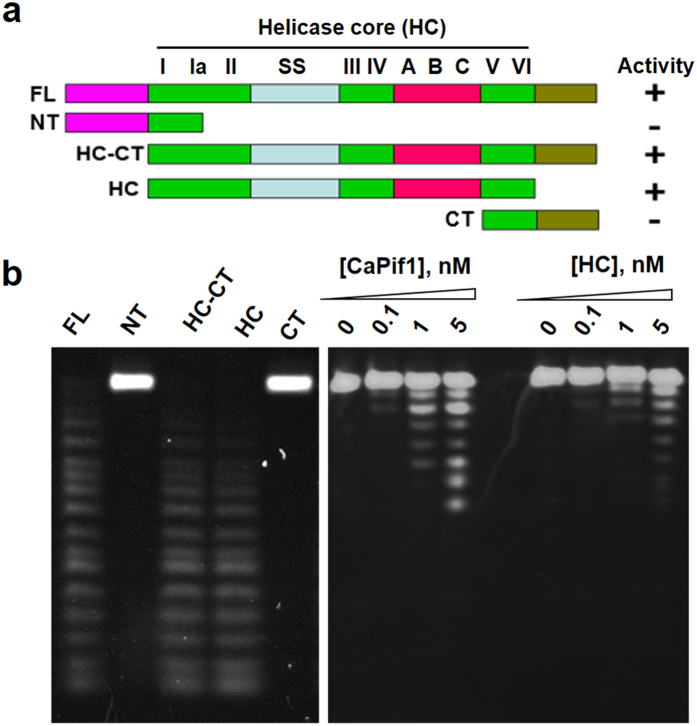
Localization of CaPif1-associated exonuclease domain. (**a**) The up panel represents the schematic summary of CaPif1 derivatives and their exonuclease activity. The sequences of the conserved SFI helicase motifs (I–VI), the Pif1/RecD-specific motifs (A–C) and the Pif1 family signature sequence (SS) are shown. NT: N-terminal domain; CT: C-terminal domain. (**b**) Exonuclease assays were performed with different domains or fragments of CaPif1 as indicated in the figure. The experiments were performed with 400 nM substrate S^20^ and 1 nM CaPif1 derivatives proteins. Comparison exonuclease activity between the full length CaPif1 and the helicase core domain (HC) of CaPif1. 400 nM substrate S^12^-5F was incubated with the protein concentration indicated for 5 min under standard exonuclease assay conditions.

CaPif1 molecule can be functionally and structurally divided into three domains (Fig. 5a, up panel): the N-terminal domain that physically and functionally interacts with yeast SSB protein to coordinate its diverse functions in cell^23^, in the human ortholog this domain exhibits a DNA strand annealing activity^24^; the highly conserved helicase core domain in various organisms shares seven conserved motifs (I, Ia, II, III, IV, V and VI) with helicases of the SF1 family as well as three additional motifs (A, B and C) that are found specifically in Pif1 and RecD family helicases^13^; the C-terminal domain is not conserved in sequence and length among different species and its function is currently unknown. To locate the exonuclease active site, we generated several CaPif1 fragments including the N-terminal domain alone (1–394aa), the N-terminal plus helicase core domain (1–878aa), helicase core domain alone (355–878aa), helicase core plus C-terminal domain (355–907aa) and C-terminal domain alone (818–907aa) (Fig. 5a). Bacterially expressed CaPif1 fragments were purified by the same protocol as described above and analyzed for exonuclease activity. Figure 5b (left panel) shows that only helicase core domain or helicase core containing fragments, but not N- or C-terminal domain alone exhibited exonuclease activity, thereby locating the CaPif1 exonucleotic activity to the helicase core domain. This conclusion was further supported by the observation in which the isolated helicase domain and the full length of CaPif1 proteins display similar exonuclease activities at a variety of the protein concentration, as judged from the digested ssDNA migrating patterns (Fig. 5b, right panel).

### Identification of the residues involved in the exonuclease active sites

Having narrowed the nuclease active sites in the helicase core domain, it was of interest to identify the precise amino acids residues implicated in the exonuclease activity. By taking the helicase core sequence as a query for sequence similarity search using BLAST program, no significant score for any identified nuclease sequences was observed in several runs. However, computational searches of the homologous Pif1 sequences from available fungal genome databases revealed the existence of 29 yeast species Pif1 proteins in which two Asp residues are highly conserved (Asp582 and Asp794 in CaPif1, Supplementary Fig. S2). Furthermore, by aligning CaPif1 sequence with several previously identified exonuclease sequences using program PHI-BLAST, we found that D582 and D794 in CaPif1 align well with the two conserved aspartic acid residues in WRN-exonuclease, Klenow fragment exonuclease (KF-exo) and DnaQ-like exonuclease domain of *A. thaliana* (Supplementary Fig. S3). More importantly, from the solved 3D structures of the exonuclesases, the conserved aspartic and glutamic residues are located in the active sites and ligate two Mg^2+^ ions directly or indirectly through water molecules^25^,^26^.

**Figure 6:**
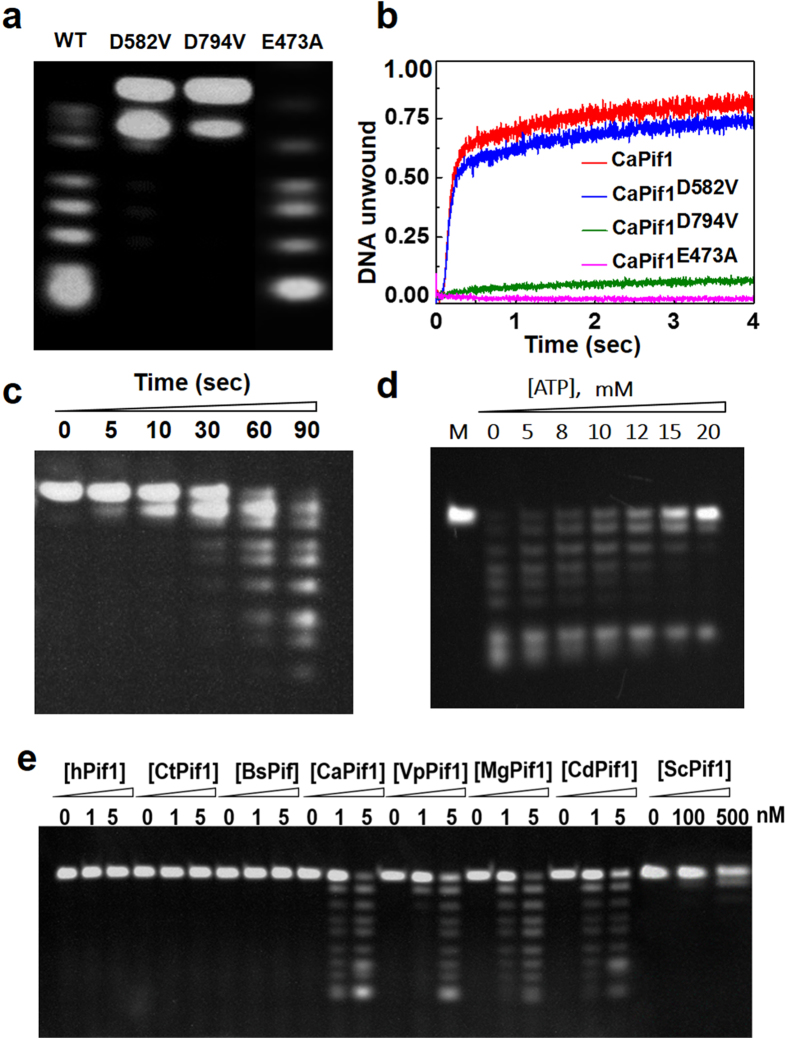
The 3′-to 5′-exonuclease activity is intrinsic to CaPif1 helicase and the interplay between the helicase and exonuclease. (**a**) Exonuclease assays with three modified CaPif1 helicases in which two aspartic acid residues were mutated into valine (D582V and D794V), one glutamic acid residue implicated in ATP hydrolysis (E473A) was mutated into alanine. The assays were performed as described in Fig. 6a. lane 1: wild type CaPif1; 2: CaPif1^D582V^; 3: CaPif1^D794V^; 4: CaPif1^E473A^. (**b**) The wild-type and mutated CaPif1 helicases-catalyzed DNA unwinding determined by stopped-flow. 4 nM 22-nt 3′-and 5′-tailed 21-bp forked DNA (substrate K) was first preincubated with 5 nM CaPif1 proteins in the reaction buffer B as described under “Materials and Methods”. The reaction was initiated by the rapid addition of 1 mM ATP and observed continuously by monitoring the fluorescence enhancement. (**c**) Kinetics of CaPif1-catalyzed DNA degradation. In this experiment, substrate J, the same sequences to substrate K used in the above stopped flow were labeled only at 5′-end. The DNA and protein concentration were 400 nM and 1 nM, respectively. (**d**) CaPif1 exonuclease activity as function of increasing ATP concentration. The substrate J was used for exonuclease assay and the DNA and protein concentrations were the same as (**c**). (**e**) The exonuclease activity assay of the different purified Pif1 helicases with increasing protein concentrations indicated in the figure. 400 nM substrate S^12^-5F was used for standard exonuclease assay.

To identify the potential aspartic acids residues implicated in the nuclease active sites of CaPif1, D582 and D794 were replaced by valine residues by site-directed mutagenesis. Analysis of the resulted mutations (CaPif1^D582V^ and CaPif1^D794V^) shows that under the same experimental condition in which the labeled ssDNA was completely digested by wild-type CaPif1, both CaPif1^D582V^ and CaPif1^D794V^ mutants displayed a strongly reduced exonuclease activity (Fig. 6a, lanes 2–3 and Supplementary Fig. S4).

### Interplay of helicase and exonuclease activities

As aforementioned, computational searches of the homologous Pif1 sequences from available fungal genome databases revealed the existence of 29 yeast species Pif1 proteins in which the above mentioned highly conserved aspartic acid residues corresponding to Asp582 and Asp794 in CaPif1 position were highly conserved except in Candida tropicalis (CtPif1) for which the equivalent Asp582 was absent. Furthermore, both conserved Asp residues were absent from human (hPif1) and from bacteria ssp (BsPif1) sequence (Supplementary Fig. S2). Therefore, it was of considerable interest to examine the potential correlation between the presence of the conserved aspartic acid residues and the observed exonuclease activity. To address the existence of a possible coordination or cross-talking between CaPif1’s helicase activity and the exonuclease activity, we prepared an additional mutant CaPif1^E473A^ in which the conserved glutamic acid 473, implicated in ATP hydrolysis, was mutated into alanine. The three mutants CaPif1^D582V^, CaPif1^D794V^ and CaPif1^E473A^ were analyzed for their helicase activities by FRET stopped-flow experiments and exonuclease activity by EMSA, respectively. We found that while the mutant CaPif1^D582V^ retained the wild-type level of helicase activity, mutant CaPif1^D794V^ exhibited a drastic reduction in exonuclease activity. In addition, mutant CaPif1^E473A^ failed to unwind duplex DNA due to the suppression of ATPase activity, as expected (Fig. 6b, pink line), but displayed the wild-type level of exonuclease activity (Fig. 6a, lane 4). These results indicated that the exonuclease activity embedded in the helicase core domain could be functionally, but not physically separable from the helicase activity under certain circumstances. Conversely, the helicase activity may be decoupled from exonuclease activity.

We then performed kinetic analysis of the CaPif1 unwinding and exonuclease activities under the same experimental conditions to determine whether two activities could be separated in the time dimension under the standard unwinding dimension. A forked duplex DNA (substrate K) (Supplementary Table S1) in which a fluorescein and a hexachlorofluorescein were labeled at the blunt end of the 5′ and 3′ extremity, respectively, was used to monitor the CaPif1 catalyzed DNA unwinding activity using FRET stopped-flow experiments (Fig. 6b). The exonuclease assay was then performed with an otherwise identical substrate (substrate J) in which only the 5′-end of the blunt end was labeled with fluorescein. Figure 6b,c show that while the DNA unwinding was completed within a time resolution of 0.5–1 s, its degradation can be only observed after 3–5 s, and 60–90 s were needed for the complete degradation. These results strongly suggested that CaPif1 first unwinds the dsDNA through its 5′-3′ helicase activity, and then digests the resulted 3′-ssDNA through its exonuclease activity.

Finally, we studied whether the exonuclease activity could be up or down-regulated by ATP that is known to be essential for helicase activity. To this end, exonuclease activity was assayed with increasing ATP concentration. Figure 6d shows that ATP did not stimulate DNA degradation, but rather significantly inhibits exonuclease activity when ATP concentration is above 12–20 mM, which is probably due to the higher product concentration (AMP) inhibits the active sites. Considering the fact that the optimum ATP concentration for DNA unwinding is 1–2 mM^27^, and that in these conditions no significant stimulatory or inhibitory effects for exonuclease activity was observed (Fig. 6d), we concluded that ATP is probably not an essential factor for the regulation of the exonuclease activity.

### The correlation between the presence of the conserved aspartic acid residues and the intrinsic exonuclease activity

According to the established phylogenetic tree (Supplementary Fig. S5), we selectively purified the Pif1 proteins containing the conserved aspartic acids from the species such as *Saccharomyces cerevisiae* (ScPif1), *Meyerozyma guilliermondii* (MgPif1), *Vanderwaltozyma polyspora* (VpPif1) and *Candida dubliniensis* (CdPif1) (Supplementary Fig. S6). Furthermore, the other Pif1 proteins in which one or both conserved aspartic acids were absent at the expected position such as hPif1, BsPif1 and CtPif1 were purified in parallel. Exonuclease activities assays have shown that while the purified CaPif1, VpPif1, MgPif1 and CdPif1 display significant exonuclease activities from 1–5 nM, hPif1, CtPif1 and BsPif1 did not display any appreciable exonuclease activities under the same experimental conditions (Fig. 6e). Furthermore, ScPif1 exhibited very weak exonuclease activity at the protein concentration as high as 500 nM (Fig. 6e and Supplementary Fig. S7). Taken together, the above observations strongly suggest that the observed exonuclease activity may be at least partially conserved in *Ascomycota subphyla*.

## Discussion

From a previous published result in which ScPif1 catalyzed telomerase disruption activity was analyzed by sequencing gels, we noted that faint bands with lower molecular weight than that of the input primers appear at the bottom of the gel^28^, suggesting that the primer or polymerized primer may be degraded by ScPif1 during the telomerase activity assay. Here, we provided biochemical evidences that some Pif1 family of helicases indeed possesses such intrinsic 3′-5′ exonuclease activity in addition to its helicase activity. This was found from the analysis of the purified Pif1 helicases from different yeast species. In particularly, we showed that the nuclease activity of CaPif1 depends on the presence of Mg^2+^ and Mn^2+^, but not Zn^2+^, Ni^2+^, Cu^2+^ and Ca^2+^. This is consistent with the observations that most exonuclease activity is mediated by metal ions^1^,^29^.

Previously, there are several reports that helicase and exonuclease activities may reside on the same polypeptide, but in distinct domains or on separate subunits to act in concert to resolve different DNA substrates formed during various DNA transactions. One well-known example is the WRN helicase that exhibits both helicase and exonuclease activities and for which the exonuclease activity resides in the N-terminal domain that is structurally autonomous and locates far from the helicase domain^25^,^30^. However it is demonstrated that the two activities could act in a coordinated fashion to remove DNA strand from long forked duplex that could not be completely unwound by the helicase activity^10^,^11^.

By characterizing several CaPif1 protein fragments, we surprisingly found that the exonuclease activity of CaPif1 could be mapped to the central helicase core, and not on the N- or C-terminus. The fact that mutations of the conserved aspartic acid residues in helicase domain abolish or strongly reduce the exonuclease activity not only eliminates the remote possibility that a bacterial exonuclease, co-purified with CaPif1 protein should be responsible for the exonucleolytic activity observed above, but also further confirm that both helicase and exonuclease activities reside on the helicase domain. Furthermore, using protein sequence analysis, we noted that D582 and D794 localize between motifs A and B, and C and V, respectively (Fig. 5), further confirming that the potential exonuclease active sites are strictly embedded in the helicase core domain, and not located in totally separated domains as found in the WRN helicase. From our knowledge, this is a first observation that the exonuclease activity is embedded in the helicase domain.

Understanding the possible biological relevance of the exonuclease activity embedded in the helicase core of Pif1 protein involved in answering three fundamental questions: first, are the helicase and exonuclease activities coupled? Second, which activity is dominant at a given condition? Third, is the exonuclease activity regulated by the concentration of ATP which is required for helicase activity? We found that while the both activities can be structurally assigned to the same domain, the two activities could be functionally separated: the elimination of the helicase activity does not abolish the exonuclease function and the reverse is true, except in the case of mutant CaPif1^D794V^, in which both activities were reduced upon the mutation, suggesting that CaPif1^D794V^ could be implicated in both helicase and exonuclease actives sites. Only the solved three dimensional structure of Pif1 in future could really clarify these questions.

More interestingly, the behavior of the coupling of helicase and nuclease activity in CaPif1 is reminiscent of the properties of RecBCD helicase. RecBCD contains one motor of each polarity: RecB bearing helicase and nuclease activity translocates along 3′-5′ direction whereas helicase subunit RecD translocates along 5′-3′ direction. Thus, two motors move along antiparallele strands with opposite unwinding polarities and at different speeds, as revealed by a loop-tail unwinding intermediates^4^. In this regard, we predict that the similar loop-tail may also exist druing CaPif-catalyzed DNA unwinding, due to the fact that the unwinding speed faster than 3′-end exonuclease speed (Supplementary Fig. S8).

The biological significance of CaPif1 exonuclease embedded in helicase core is not yet known, many questions remain to be answered: whether and how both activities contribute to the same biological process in a coordinated manner? What is the structural basis of the helicase core domain possessing an exonuclease activity? The future genetic and structural biological studies will provide insight into our uncovered phenomenon.

## Methods

### Cloning procedures

The genes encoding for different Pif1 helicases were amplified by PCR using genomic DNAs from the corresponding strains collected by CGMCCC (China General Microbiological Culture Collection Center) except human Pif1 and Yeast Pif1, which are gifts from Dr. Sanders CM and Zakian VA. The resulted DNA fragments were cloned into pET15b-SUMO (Invitrogen) using ExTaq PCR (Takara) according to the manufacturer’s protocol. The entire inserts were sequenced, and all of the mutations revealed by comparison with the Genome Database (http://www.ncbi.nlm.nih.gov/genome/) were replaced. Site-directed mutagenesis was performed by overlap extension PCR method as described previously^31^. The PCR primers used in mutation studies were summarized in Supplementary Table S2.

### Protein expression and purification

Human Pif1 and ScPif1 proteins were exactly purified according to the published protocols^32^,^33^. The other Pif1 proteins used in this work were overexpressed in *Escherichia coli* BL21 (pLysS) at 18 °C for 16 h. The cells were lysed by sonication in 20 mM Tris-HCl at pH 7.6, 500 mM NaCl, 5 mM imidazole, and 10% Glycerol on ice. The protein was purified over a Ni-NTA column followed by SUMO protease cleavage of the hexahistidine tag overnight at 4 °C. The cleaved proteins–proteases mixtures were dialyzed to remove the excess imidazole and the protein was further purified over a second Ni-NTA column that was used to remove all His-tagged products. The Ni-NTA flow through was dialyzed against buffer A (20 mM Tris-HCl at pH 7.6, 100 mM NaCl, 1 mM DTT and 10% Glycerol) and was then loaded on a Source SP column (GE Healthcare) and eluted in gradient (100–1000 mM NaCl). The proteins were further purified by a Mono Q column to remove any traces of protein contaminants. At this stage the proteins were more than 95% pure. The proteins were finally purified over a superdex-S200 sizing column pre equilibrated with 50 mM Tris-HCl, pH 7.5, 10% glycerol, 0.5 M KCl and 1 mM DTT, and the proteins were concentrated to ≈10 mg/ml using an Amicon 30 K cutoff (Millipore) and stored at 4 °C for subsequent studies.

### DNA substrates and fluoroscein labeling

All DNA substrates used in this study were purchased from Shanghai Sangon Biological Engineering Technology & Services Co., Ltd (Shanghai, China) and the sequences were summarized in Supplementary Table S1. The fluorescein or hexachlorofluorescein labeling oligos were used for monitor helicase or/and exonuclease activities as described below. To generate different structured DNAs used in the nuclease assays, the 3′-or 5′-fluorescein labeled DNAs were incubated with the unlabeled complementary strands in a hybridization buffer containing 20 mM Tris-HCl pH 7.5 and 100 mM NaCl. The mixture was incubated at 95 °C for 10 min and then left to stand at room temperature about 4 h. The resulted DNAs were further purified by a Mono Q column with NaCl gradient elution.

### Nuclease assay

The proteins were incubated with appropriate various fluorescein labeled substrate DNAs in a standard reaction mixture in buffer A (40 mM Tris-HCl, pH 7.8, 100 mM NaCl, 20 mM KCl, 2 mM MgCl_2_, 2 mM Mg(AC)_2_) at 37 °C. Reaction time, DNA and protein concentrations are described in each figure legend. The reactions were stopped with 2X loading buffer (8 M urea, 0.05%Xylene Cyanole) and then heated for 5 min at 98 °C. These samples were subjected to 15% polyacrylamide-8M urea gel electrophoresis, and the degradation products were visualized by Gel imaging analysis system (GeI Doc-It310, UVP, America).

### Helicase Assay

We used a stopped-flow FRET assay for measuring unwinding activity of CaPif1 and its mutants. DNA substrates were labeled with fluorescein and hexachlorofluorescein as a donor and acceptor, respectively. The stopped-flow FRET assay was carried out using a Bio-Logic SFM-400 mixer with a 1.5 mm × 1.5 mm cell (FC-15, Bio-Logic, France) and the Bio-Logic MOS450/AF-CD optical system equipped with a 150-watt mercury-xenon lamp. Fluorescein was excited at 492 nm (2-nm slit width), and its emission was monitored at 525 nm using a high pass filter with 20-nm bandwidth (D525/20; Chroma Technology Co.), unless noted elsewhere. Unwinding kinetics were measured in a two-syringe mode, where the helicases and DNA substrates were pre-incubated in the unwinding buffer B (25 mM Tris-HCl pH 7.5, 50 mM NaCl, 2 mM MgCl_2_, 1 mM DTT) at 25 °C for 5 min (in syringe 3) and the unwinding reaction was initiated by rapid mixing ATP (in syringe 4). All concentrations listed correspond to final concentrations, i.e. after mixing. For converting the output data from volts to percentage of unwinding, a calibration experiment was performed in a four-syringe mode, where helicase in syringe 1, hexachlorofluorescein-labeled single-stranded oligonucleotides in syringe 2, and fluorescein-labeled single-stranded oligonucleotides in syringe 3 were incubated in unwinding reaction buffer, and ATP with or without protein trap were in syringe 4. The fluorescent signal of the mixed solution from the four syringes corresponded to 100% unwinding. The stopped-flow temperature was controlled by an external thermostated water bath (Ministat 125; Huber) and a high flux pump.

#### Kinetic Data Analysis

All stopped flow kinetic traces were an average of over 10 individual traces. The kinetic traces were analyzed using Bio-Kine (version 4.26; Bio-Logic, France).

### Bioinformatic procedures

Search for the homologous sequences in the databases with BLAST was carried out at a website (http://blast.ncbi.nlm.nih.gov/Blast.cgi). The sequence alignments with solved 3D structure exonucleases including human Werner syndrome protein, *E. coli* polymerase I and Arabidopsis thaliana DnaQ-like exonuclease was carried out using PSI- and PHI-BLAST and was further manually refined. The other sequences were picked up from the database. The accession numbers were summarized in the Supplementary Table S3.

## Additional Information

**How to cite this article:** Wei, X.-B. *et al*. A 3′-5′ exonuclease activity embedded in the helicase core domain of *Candida albicans* Pif1 helicase. *Sci. Rep.* **7**, 42865; doi: 10.1038/srep42865 (2017).

**Publisher's note:** Springer Nature remains neutral with regard to jurisdictional claims in published maps and institutional affiliations.

## Supplementary Material

Supporting Information
