# Supplementary material for: A 3′-5′ exonuclease activity embedded in the helicase core domain of Candida albicans Pif1 helicase
Source: Sci Rep. 2017 Feb 20;7:42865. doi: 10.1038/srep42865 (PMC5316945; doi:10.1038/srep42865)
Supplement: Supporting Information [file srep42865-s1.pdf]

Supporting Information

A 3'-5' exonuclease activity embedded in the helicase core domain of *Candida albicans* Pif1 helicase

Xiao-Bin WEI[1§], Bo ZHANG[1§], Nicolas BAZEILLE[2§], Ying YU[1], Na-Nv LIU[1], Brigitte RENE[2], Olivier MAUFFRET[2], Xu-Guang XI[1,2,*]

[1]College of Life Sciences, Northwest A&F University, Yangling, Shaanxi 712100, China

[2]Laboratoire de Biologie et Pharmacologie Appliquée, Ecole Normale Supérieure de Cachan, Université Paris-Saclay, Centre National de la Recherche Scientifique, 61 Avenue du Président Wilson, 94235 Cachan, France

*Corresponding authors: email: xxi01@ens-cachan.fr
Tel: 33 01 4740 7754; Fax: 33 01 4740 7754;

[§]These authors contributed equally to this work.

Table S1. substrates used for activities assays

Name	Length (nt)	Structure	Sequences (5'-3')
S^{20}	20	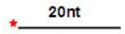	*TTGATTTGATGTACGGATGT
Bb^{4}	40/40	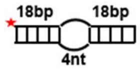	*GCAGTAGGCCTAGCATGAGTTAGGACTGACAGCTGCATGG<br>CCATGCAGCTGTCAGTCCATTGTCATGCTAGGCCTACTGC
Bb^{12}	40/40	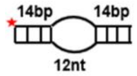	*GCAGTAGGCCTAGCATGAGTTAGGACTGACAGCTGCATGG<br>CCATGCAGCTGTCACAGGATTGAGTAGCTAGGCCTACTGC
S^{12}-5F	12	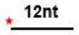	*GTACGGATGTCT
S^{12}-3F	12	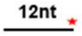	GTACGGATGTCT*
$S^{20}D^{23}$-5F	43/23	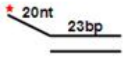	*ATGTTACATTTATTTTGTAGCAAAAATAAAGTGTAAGGATGTC<br>GACATCCTTACACTTTATTTTTG
$S^{20}D^{23}$-3F	43/23	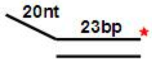	ATGTTACATTTATTTTGTAGCAAAAATAAAGTGTAAGGATGTC*<br>GACATCCTTACACTTTATTTTTG
A	23	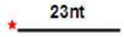	*GTGTAAGGATGTCACGATCATTG
B	23/23	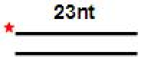	*GTGTAAGGATGTCACGATCATTG<br>CAATGATCGTGACATCCTTACAC
C	23/22	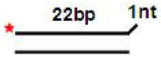	*GTGTAAGGATGTCACGATCATTG<br>AATGATCGTGACATCCTTACAC
D	23/21	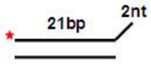	*GTGTAAGGATGTCACGATCATTG<br>ATGATCGTGACATCCTTACAC
E	43/30	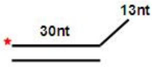	*ATGTTACATTTATTTTGTAGCAAAAATAAAGTGTAAGGATGTC<br>TTTATTTTTGCTACAAAATAAATGTAACAT
F	43/25	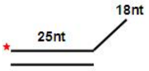	*ATGTTACATTTATTTTGTAGCAAAAATAAAGTGTAAGGATGTC<br>TTTTGCTACAAAATAAATGTAACAT
G	23/43	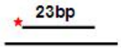	*GTGTAAGGATGTCACGATCATTG<br>TTTTTTTTTTCAATGATCGTGACATCCTTACACTTTTTTTTTT
H	23/43	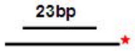	CACTTTATTTTTGCTACAAAATA<br>*ATGTTACATTTATTTTGTAGCAAAAATAAAGTGTAAGGATGTC
I	43/21	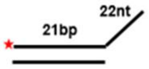	*ATGTTACATTTATTTTGTAGCAAAAATAAAGTGTAAGGATGTC<br>GCTACAAAATAAATGTAACAT
J	43/43	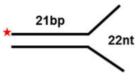	*ATGTTACATTTATTTTGTAGCAAAAATAAAGTGTAAGGATGTC<br>CTGTAGGAATGTGAAATAAAAAGCTACAAAATAAATGTAACAT
K	43/43	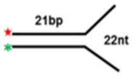	*ATGTTACATTTATTTTGTAGCAAAAATAAAGTGTAAGGATGTC<br>CTGTAGGAATGTGAAATAAAAAGCTACAAAATAAATGTAACAT*

*, Fluorescein

*, hexachlorofluorescein

Table S2. Primers used for mutagenesis

Name	Sequence (5'-3')
CaPif1^{D582V}-F	GACGACACCGCGCGTGTATTCTGCCGTCTGTCT
CaPif1^{D582V}-R	AGACAGACGGCAGAACTCACGCGCGGTGTCGTC
CaPif1^{D794V}-F	CAGTGGACCACCGAAGTTGAAGACGGTACCGTT
CaPif1^{D794V}-R	AACGGTACCGTCTTCAACTTCGGTGGTCCACTG
CaPif1^{E473A}-F	GTTCTGATCATCGACGCGATCTCTATGGTTGAC
CaPif1^{E473A}-R	GTCAACCATAGAGATCGCGTCGATGATCAGAAC

Table S3. Proteins accession numbers for sequence alignment

**Orgnism**	**Strain**	**Accession numbers**
Naumovozyma dairenensis	CBS421	XP_003670036.1
Naumovozyma castellii	CBS4309	XP_003673236.1
Saccharomyces cerevisiae	S288c	NP_013650.1
Kazachstania africana	CBS2517	XP_003958958.1
Candida glabrata	CBS138	XP_449255.1
Zygosaccharomyces rouxii	CBS732	XP_002495556.1
Torulaspora delbrueckii	CBS1146	XP_003680891.1
Vanderwaltozyma polyspora	DSM70294	XP_001643959.1
Tetrapisispora phaffii	CBS4417	XP_003684156.1
Tetrapisispora blattae	CBS6284	XP_004181037.1
Ashbya gossypii	ATCC10895	NP_984987.1
Eremothecium cymbalariae	DBVPG#7215	XP_003644227.1
Kluyveromyces lactis	NRRL Y-1140	XP_451763.1
Lachancea thermotolerans	CBS6340	XP_002553410.1
Millerozyma farinosa	CBS7064	XP_004197165.1
Debaryomyces hansenii	CBS767	XP_459513.2
Meyerozyma guilliermondii	ATCC6260	XP_001484923.1
Lodderomyces elongisporus	NRRL YB-4239	XP_001523552.1
Candida orthopsilosis	Co90-125	XP_003870993.1
Candida tropicalis	MYA-3404	XP_002548760.1
Candida albicans	SC5314	XP_712376.1
Candida dubliniensis	CD36	XP_002422246.1
Komagataella pastoris	GS115	XP_002491346.1
Emiliania huxleyi virus	86	YP_293894.1
Homo sapiens		NP_079325.2

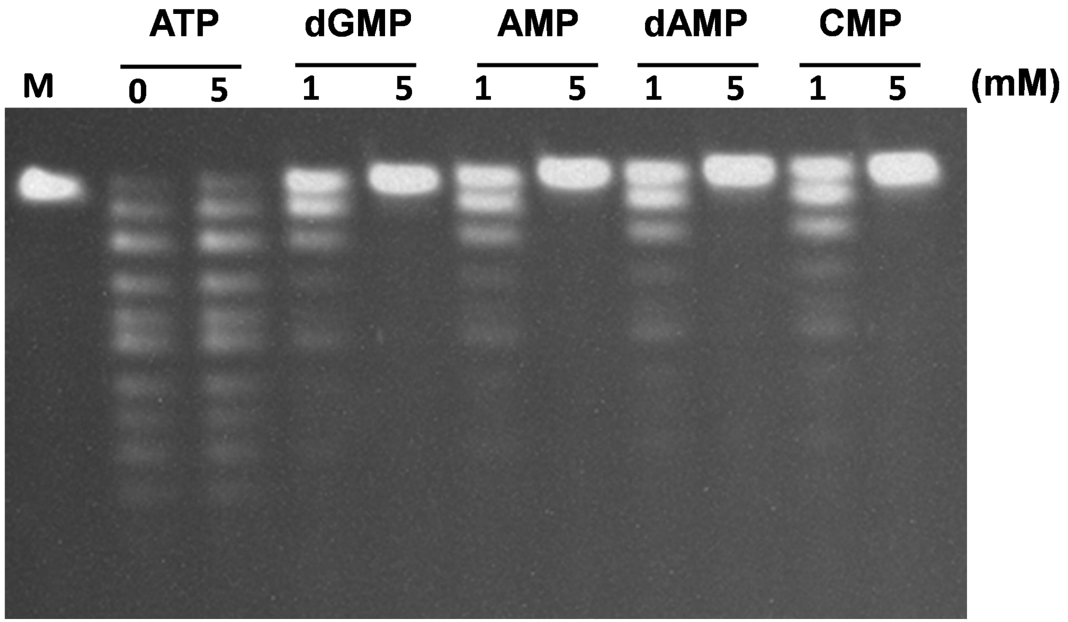

Figure S1. CaPif1 exnoclease activity was inhibited by nucleotide monophosphates. The experiments were performed under the standard exonuclease assay conditions with substrate S12-5F.

Strains		Conserved E/D	Conserved E/D
C.albicans	SC5314	572 DGNVPDDTARDFCRLSRPLKC 592	785 VVEPEQWTTEDEDGT-VLVSR 804
C.dubliniensis	CD36	573 DGNVPDDTVRDFCRLSRPLKC 593	786 VVEPEQWTTEDDEGK-VLVSR 805
C.tropicalis	MYA-3404	512 DGMINKESIVRFQQLERPLKC 532	685 IVDVESWSTEDVETESILAKR 705
L.elongisporus	NRRL YB-4239	715 DGRISDRTVREFQRLSRPLSC 735	927 LVEPEEWTVEDEDGH-VLVSR 946
C.orthopsilosis	Co90-125	622 DGRISESTIQEFRRLSRPLEC 642	821 LVEPEQWTIEDEDGR-ALVSR 840
M.farinosa	CBS7064	428 SGKVTSDTIAEFNKLTRPLET 448	654 LVEPEEWTVEDEHEN-VLVRR 673
D.hansenii	CBS767	480 NGKVNNETIAEFKRLSRKLDN 500	707 LVEPEQWTIEDENEN-VLARR 726
M.guilliermondii	ATCC6260	368 SGQIGPDTEMEFKKLARKLEC 388	596 LVEPETWTVEDEHQQ-VLASR 615
N.dairenensis	CBS421	473 LGKIDENTEREFKKLTRPLA- 492	706 LVEPENWAIEDENEK-PIVSR 725
N.castellii	CBS4309	475 LGKIDDETEREFRKLSRSLP- 494	709 LVEPECWEIEDENSK-PIVSR 728
Z.rouxii	CBS732	441 LGQIDEDTEMEFKRLNRPLP- 460	678 LVEPEDWAIEDENEK-PLVSR 697
T.delbrueckii	CBS1146	526 LGQIDAETEREFKKLNRPLP- 545	764 LVEPESWAIEDDNEK-SLVSR 783
K.africana	CBS2517	418 LGQIDEDTEREFKKLSRPLP- 437	654 LVEPEDWAIEDEHEK-PIVSR 673
S.cerevisiae	S288c	433 LGNIDDETEREFKKLSRPLP- 452	672 LVEPEDWAIEDENEK-PLVSR 691
V.polyspora	DSM70294	553 LGEIDIETETEFKKLSRELE- 572	791 LVEPEDWSIEDENEK-PIVSR 810
C.glabrata	CBS138	455 LGKIDMETEMEFKKLNRPLP- 474	690 LVEPEDWAIEDENEK-PLVSR 709
T.phaffii	CBS4417	547 VGNVDSDTEMEFKKLSRPLP- 566	787 LVEPEDWAIEDEDEK-PIVSR 806
T.blattae	CBS6284	442 LGKLDFKMEIEFKKLQRPLP- 461	678 LVEPEDWSVEDENEK-PIVSR 697
A.gossypii	ATCC10895	393 LGKISEETEAEFRKLSRPLV- 412	634 LVREEAWAIEDENEK-PLVTR 653
E.cymbalariae	DBVPG#7215	474 LGKISPETELEFRKLSRPLE- 493	715 LVREEAWAVEDENEK-PLVTR 734
K.lactis	NRRL Y-1140	454 MGVISDRTLKEFAELSRPLP- 473	696 LVRPETWAMEDEKDV-PLVSR 715
L.thermotolerans	CBS6340	488 MGKISLETELEFKKLSRPLP- 507	723 LVQPEDWAVEDELQK-PIVSR 742
K.pastoris	GS115	427 NGRISPRVEAEFQKLNRPLPV 447	655 LVEPEQFSINDIDEK-PLASR 674
E.huxleyi virus	86	196 IGKCATETENALAKCHVSVKP 216	327 VMQMERAETEQKFMN-SKCTR 346
H.sapiens		396 LGRCSDEVTRQLQATASHKVG 416	523 VIHADRWTVQATGGQ--LLSR 542

Figure S2. Sequence alignment of selected fungal species using BLAST. The accession numbers of the proteins sequences were summarized in the Supplementary Table S3.

C.albicans	*pif1*	563	FIDMLNNLRDGNVPDDTARDFCRLSRPLKC-PEG	595
H.sapiens	*WRN-exo*	124	LKMLLENKAVKKAGVGIEGDQWKLLRDFDIKLKN	157
E.coli	*KF-exo*	405	LKPLLEDEKALKVGQNLKYDRGILAN-YGIELRG	437
A.Thaliana	*1VK0*	100	LYRFFASKFVTFVGVQIEEDLD-LLRENHG-LVI	131
C.albicans	*pif1*	776	DGITFRTVVVE-PEQWTTEDEDGTVLVSRIQFPL	808
H.sapiens	*WRN-exo*	197	CSNWSKFPLTEDQKLYAATDAYAGFIIYRNLELL	230
E.coli	*KF-exo*	483	QLTFNQIAL-EEAGRYAAEDADVTLQLHLKMWPD	515
A.Thaliana	*1VK0*	173	IEAKWEKAGPEEQLEAAAIEGWLIVNVWDQLSDE	206

Figure S3. Sequence alignment of CaPif1 with WRN-exo, Kf-exo and *A. thaliana* homolog was carried out using PHI-BLAST. The numbers between the sequences indicate the amino-acid position relative to the amino terminus of each protein and the conserved aspartic/glutamic residues were colored in red.

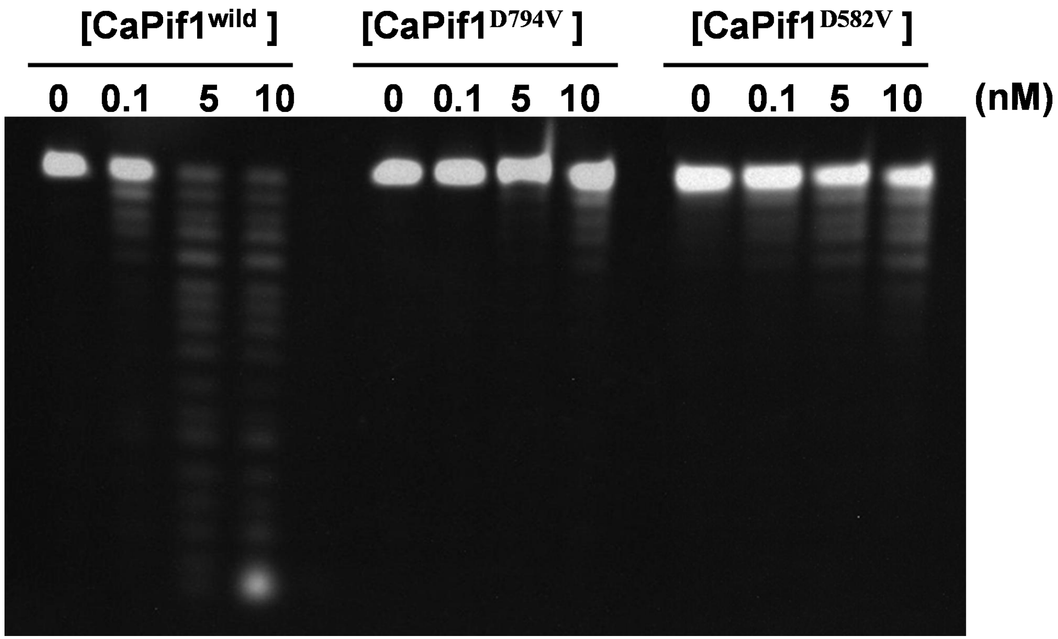

Figure S4. Gel electrophoresis measurements of exonuclease activity of wild-type CaPif1 , mutants CaPif1^{D582V} and CaPif1^{D794V} purified proteins. The reactions were carried out at 37°C for 5min in buffer A with 400nM 5'-fluorescein labeled 20-nt ssDNA (substrate S^{20}) at the indicated protein concentrations.

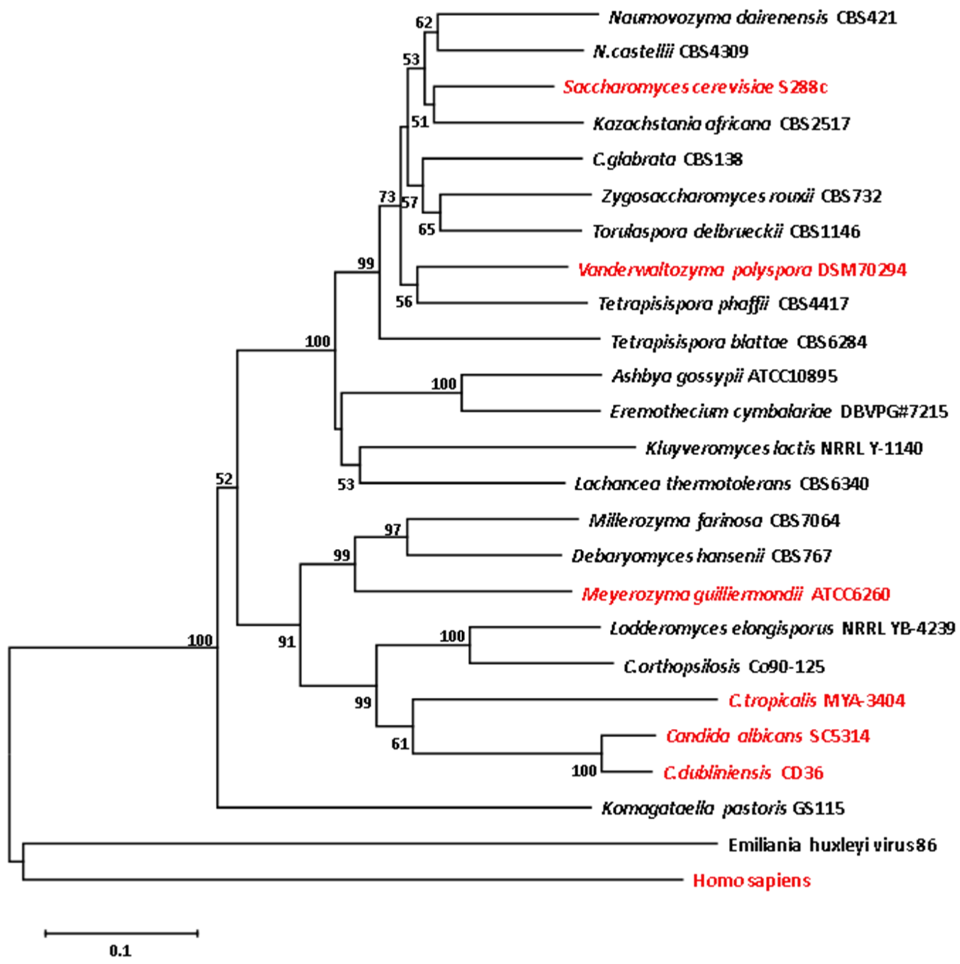

Figure S5. Selected Pif1 helicases from the constructed phylogenetic tree were used for confirming the relationship between the presence of the conserved aspartic/glutamic residues and exonuclease activities. The protein sequences accession numbers are summarized in Table S3.

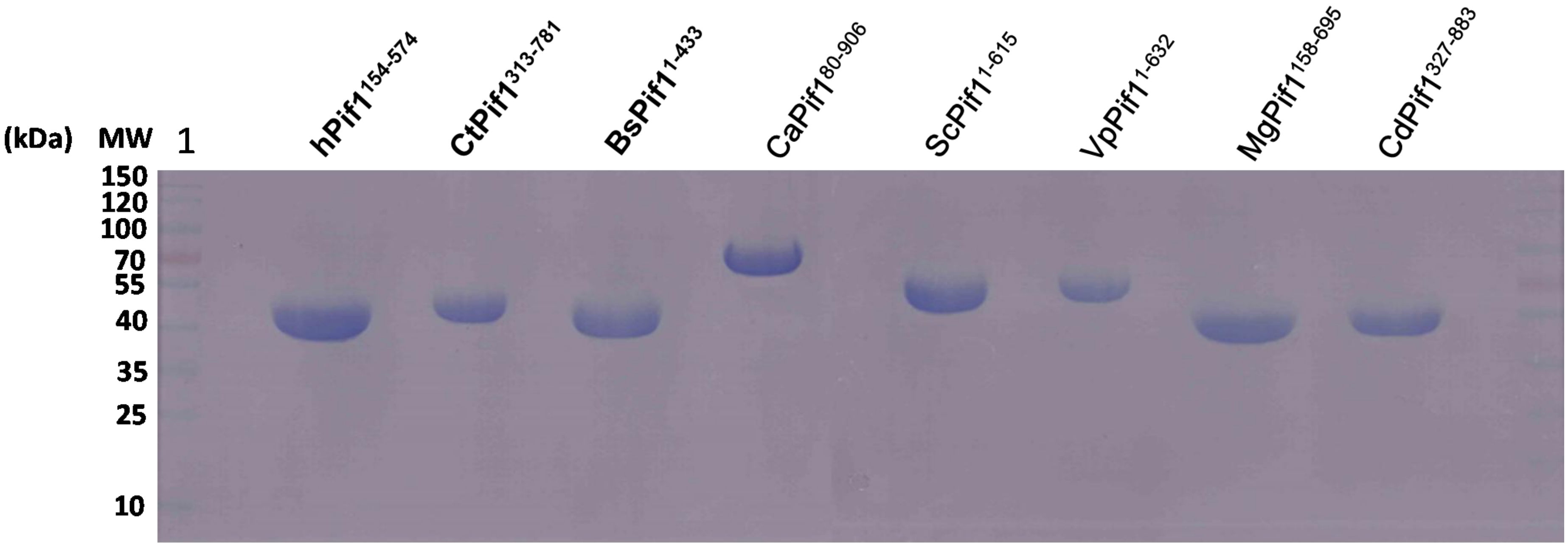

Figure S6. Different kinds of pif1 protein purity. SDS-PAGE analysis of the purified 8 kinds of Pif1 protein. The gel (10% acrylamide) was stained using Coomassie brilliant blue R250. Lane 1 show protein molecular maker and all the protein (20µg), respectively.

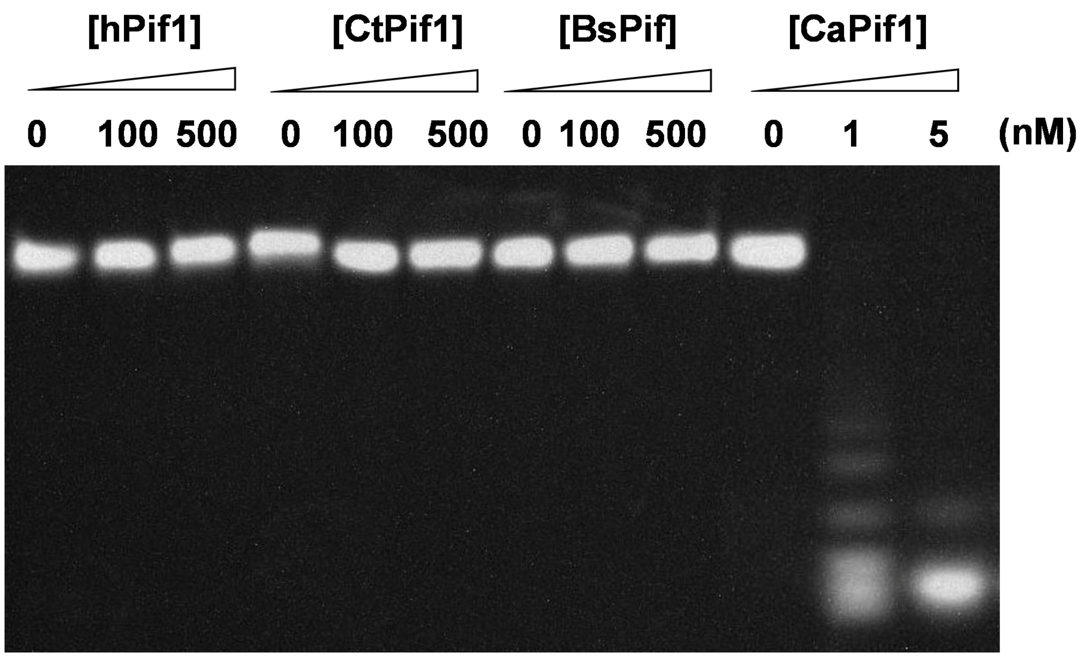

Figure S7. hPif1, BsPif1 and CtPif1 do not display any appreciable exonuclease activities when high concentration of protein was added. The experiments were performed under the standard exonuclease assay conditions with substrate S12-5F.

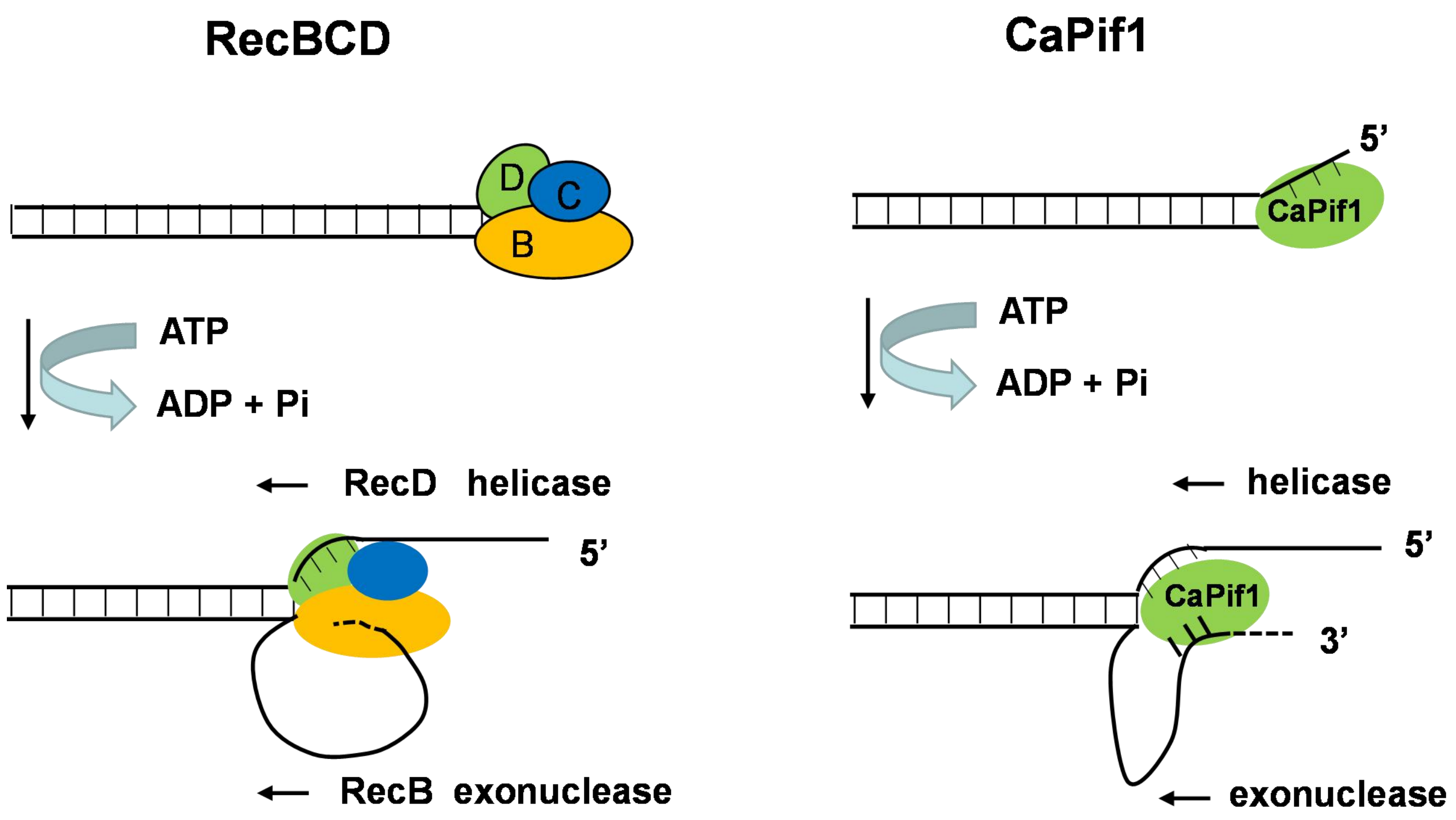

Figure S8. In accordance to the properties of RecBCD helicase, we predict the model that coupling of helicase and nuclease activity in CaPif1.
